# Synthesis, crystal structure and Hirshfeld surface analysis of 7-oxo-6-phenyl-6,7-di­hydro-5*H*-thieno[2,3-*f*]iso­indole-8-carb­oxy­lic acid

**DOI:** 10.1107/S2056989025008618

**Published:** 2025-10-07

**Authors:** Evgeniya R. Shelukho, Vladimir P. Zaytsev, Victor N. Khrustalev, Tuncer Hökelek, Khudayar I. Hasanov, Narmina A. Guliyeva, Tahir A. Javadzade, Mohammed Hadi Al-Douh

**Affiliations:** a RUDN University, 6 Miklukho-Maklaya Str., 117198 Moscow, Russian Federation; b Zelinsky Institute of Organic Chemistry of RAS, Leninsky Prospect 47, 119991 Moscow, Russian Federation; c Hacettepe University, Department of Physics, 06800 Beytepe-Ankara, Türkiye; d Azerbaijan Medical University, Scientific Research Centre (SRC), A. Kasumzade Str. 14, AZ1022, Baku, Azerbaijan; e Baku Engineering University, Khirdalan, Hasan Aliyev Str. 120, AZ0101 Absheron, Azerbaijan; f Department of Chemistry and Chemical Engineering, Khazar University, Mahsati Str. 41, AZ1096, Baku, Azerbaijan; g Chemistry Department, Faculty of Science, Hadhramout University, Mukalla, Hadhramout, Yemen; Universidade de Sâo Paulo, Brazil

**Keywords:** intra­molecular dide­hydro-Diels–Alder reaction, propargyl­amine, IMDDA reaction, thieno[2,3-*f*]iso­indole, crystal structure

## Abstract

In the title compound, C_17_H_11_NO_3_S, the thieno[2,3-*f*]iso­indole ring system and the phenyl ring are oriented at a dihedral angle of 20.57 (13)°. The strong intra­molecular O—H⋯O hydrogen bond partly ensures the coplanarity of the carboxyl group and the ring system. In crystal, the mol­ecules are linked through C—H⋯O hydrogen bonds, enclosing *R*^2^_2_(14) ring motifs, into a three-dimensional architecture. π–π inter­actions between parallel five-membered and phenyl rings [centroid-to-centroid distances of 3.564 (3) and 3.591 (3) Å] further contribute to the cohesion of the crystal structure. The Hirshfeld surface analysis indicates that the most important contributions for the crystal packing are from H⋯H (35.5%), H⋯O/O⋯H (21.3%), C⋯C (14.1%) and H⋯C/C⋯H (12.8%) inter­actions.

## Abstract 2

The mol­ecule of the title compound contains a planar thieno[2,3-*f*]iso­indole ring system and a phenyl ring. In crystal, the mol­ecules are linked through C—H⋯O hydrogen bonds, enclosing *R*^2^_2_(14) ring motifs, into a three-dimensional architecture. π–π inter­actions further consolidate the crystal structure.

## 1. Chemical context

It is widely recognized that in the field of [4 + 2]-cyclo­addition chemistry, the terms diene and dienophile are not limited to compounds with only double bonds. In the Diels–Alder reaction, alkynes can also serve as dienophiles, and conjugated 1,3-diynes or 1,3-enynes can act as dienes. Such pericyclic reactions are referred in the literature as de­hydro-Diels–Alder reactions (Wessig *et al.*, 2008 ▸; Johnson, 2010 ▸; Ajaz *et al.*, 2011 ▸; Li *et al.*, 2016 ▸), and intra­molecular de­hydro-Diels–Alder (IMDDA) reactions are widely used in organic synthesis to construct polycyclic mol­ecules (Hoye *et al.*, 2012 ▸; Brummond *et al.*, 2015 ▸; Wang *et al.*, 2016 ▸; Rana *et al.*, 2017 ▸; Krishna *et al.*, 2022 ▸). A special case of this type of transformation is the intra­molecular dide­hydro-Diels–Alder reaction, in which an alkene dienophile reacts with an enyne. There are only a few publications related to the intra­molecular de­hydro-Diels–Alder reaction of the thio­phene series that demonstrate the fundamental possibility of IMDDA transformations (Klemm *et al.*, 1965 ▸, 1966 ▸; Lu *et al.*, 2005 ▸; Bober *et al.*, 2017 ▸; Huang *et al.*, 2017 ▸).
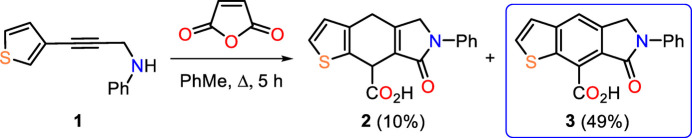


This work is a continuation of studies on the tandem acyl­ation/[4 + 2] cyclo­addition reaction between 3-(thien­yl)propargyl­amines and maleic anhydride as an example of the IMDDA approach (Shelukho *et al.*, 2025 ▸). Thienylpropargyl­amine **1** readily reacts with maleic anhydride to provide a mixture of products, where the product **2** has been published previously (Shelukho *et al.*, 2025 ▸), but the major acid **3** could not be isolated and characterized due to the formation of a mixture of products. In this work, we successfully isolated and characterized 7-oxo-6-phenyl-6,7-di­hydro-5*H*-thieno[2,3-*f*]iso­indole-8-carb­oxy­lic acid, **3**. The detection of acid **3** directly confirms the assumption that type **2** di­hydro­acid is prone to easy oxidation under aerobic conditions. Herein, we report the synthesis and mol­ecular and crystal structure, together with the Hirshfeld surface analysis of the title compound, **3**.

## 2. Structural commentary

**Figure 1:**
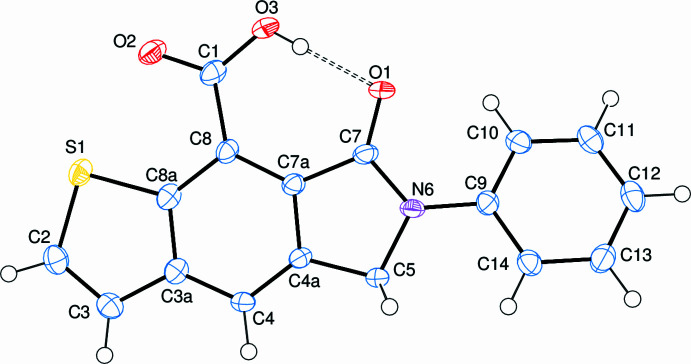
The asymmetric unit of the title compound with the atom-numbering scheme and 50% probability ellipsoids.

The asymmetric unit of the title compound, C_17_H_11_NO_3_S, contains an essentially planar [r.m.s. deviation = 0.02 (4) Å] thieno[2,3-*f*]iso­indole ring system (*A;* S1/C2/C3/C3*A*/C4/C4*A*/C5/N6/C7/C7*A*/C8/C8*A*), and a phenyl ring (*B*; C9–C14) oriented at a dihedral angle of 20.57 (13)°. The carboxyl group (C1/O2/O3/C8) is oriented at a dihedral angle of 0.22 (12)° with respect to the thieno[2,3-*f*]iso­indole ring system. Thus, they are almost coplanar, partly as a result of the strong intra­molecular O3—H3*O*⋯O1 hydrogen bond between the carboxyl hydrogen and iso­indole oxygen atoms (Table 1 ▸, Fig. 1 ▸). On the other hand, the dihedral angle between the carboxyl­ate group and ring *B* is 21.2 (4)°. Atom O1 is 0.037 (3) Å away from the best least-squares plane of the thieno[2,3-*f*]iso­indole ring system. In the carboxyl­ate group, the O2—C1 and O3—C1 bond lengths are 1.217 (6) and 1.303 (6) Å, respectively. Thus, the C—O bonds in the carboxyl­ate group indicate mainly localized single and double bounds rather than a delocalized bonding arrangement. The O2—C1—O3 [120.9 (4)°] bond angle seems to be decreased compared to that present in a free acid (122.2°; Sim *et al.*, 1955 ▸) and compares with corresponding values of 122.42 (14)° in C_17_H_13_NO_2_ (Refcode UYATIZ; Mague *et al.*, 2016 ▸), 122.55 (12)° in C_14_H_11_NO_3_ (BIYJEC; El-Mrabet *et al.*, 2023 ▸) and 122.70 (12)° in C_12_H_10_ClNO_3_ (PEDKAO; Filali Baba *et al.*, 2022 ▸). As indicated by the O2—C1—C8—C7*A* [179.9 (4)°] and O3—C1—C8—C8*A* [179.7 (4)°] torsion angles, the carboxyl group attached to the thieno[2,3-*f*]iso­indole ring system is in a *anti* peripheral conformation.

## 3. Supra­molecular features

**Figure 2:**
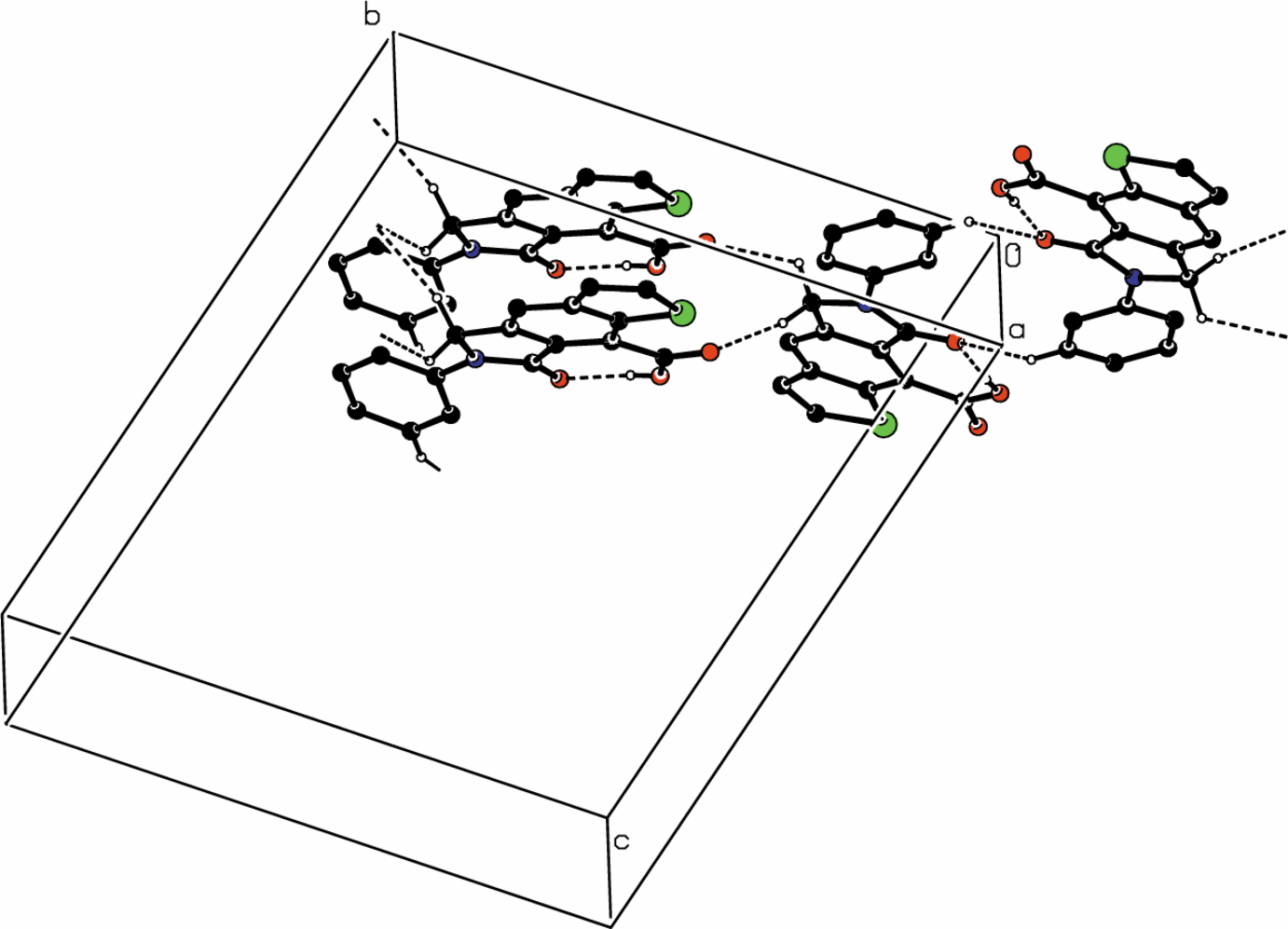
A partial packing diagram of the title compound viewed down the *a*-axis direction. Intra­molecular O—H⋯O and inter­molecular C—H⋯O hydrogen bonds are shown as dashed lines. H atoms not involved in these inter­actions have been omitted for clarity.

In the crystal, the mol­ecules are linked through C—H⋯O hydrogen bonds, enclosing 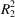
(14) ring motifs, into a three-dimensional architecture (Fig. 2 ▸). There are π–π inter­actions between the parallel five-membered (S1/C2/C3/C3*A*/C8*A* and N6/C5/C4*A*/C7*A*/C7) rings and the phenyl (C3*A*/C4/C4*A*/C7*A*/C8/C8*A*) ring with centroid-to-centroid distances of 3.564 (3) Å (α = 0.92° and slippage = 1.031 Å) and 3.591 (3) Å (α = 0.70° and slippage = 1.041 Å), respectively.

## 4. Hirshfeld surface analysis

**Figure 3:**
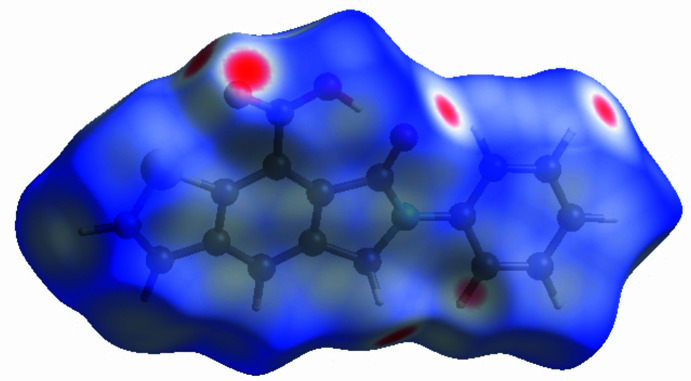
View of the three-dimensional Hirshfeld surface plotted over *d*_norm_.

**Figure 4:**
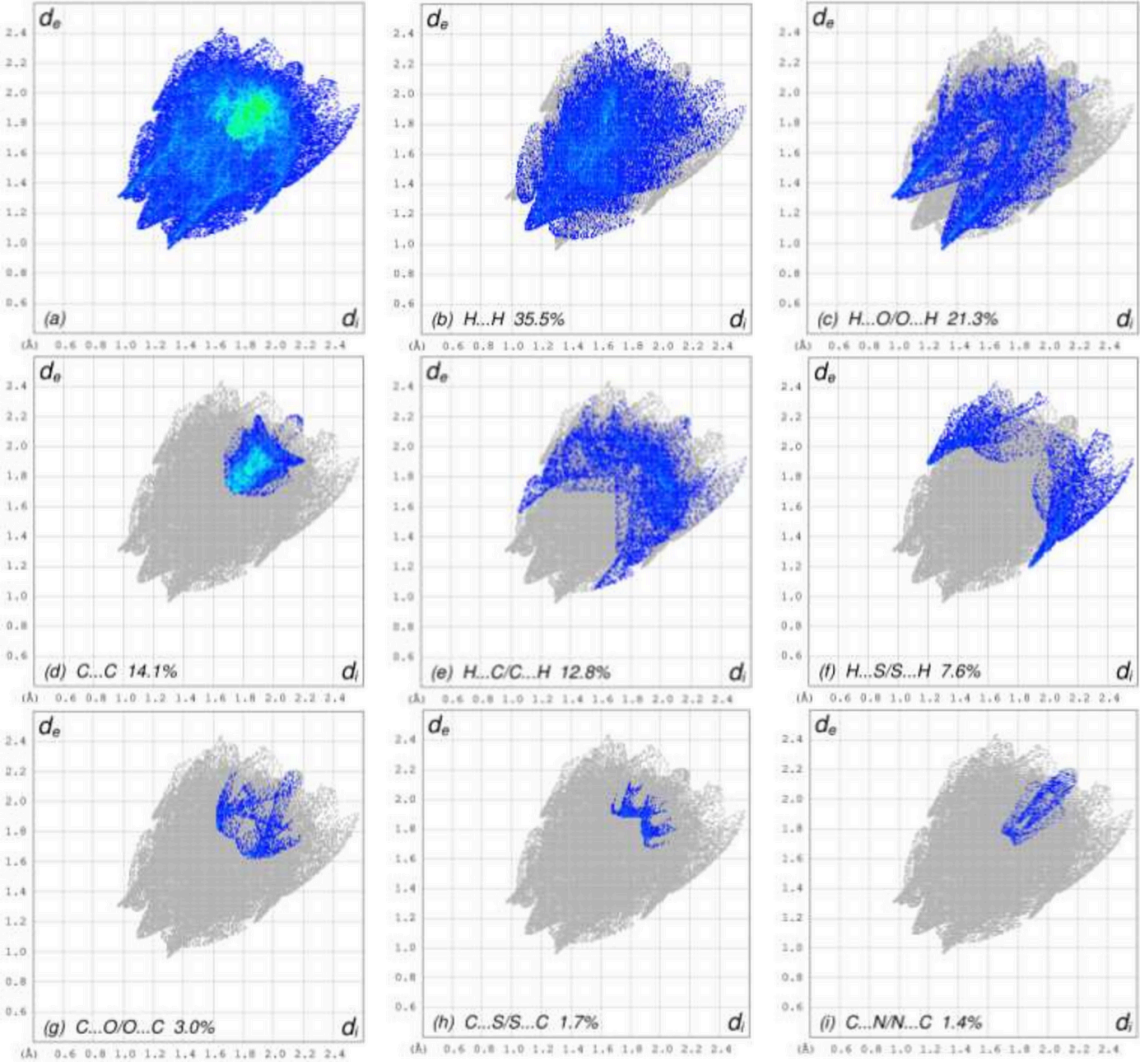
The full two-dimensional fingerprint plots showing (*a*) all inter­actions, and delineated into (*b*) H⋯H, (*c*) H⋯O/O⋯H, (*d*) C⋯C, (*e*) H⋯C/C⋯H, (*f*) H⋯S/S⋯H, (*g*) C⋯O/O⋯C, (*h*) C⋯S/S⋯C, (*i*) C⋯N/N⋯C, (*j*) O⋯S/S⋯O, (*k*) O⋯O, (*l*) H⋯N/N⋯H, (*m*) S ⋯ S and (*n*) N⋯N inter­actions. The *d*_i_ and *d*_e_ values are the closest inter­nal and external distances (in Å) from given points on the Hirshfeld surface.

To visualize the inter­molecular inter­actions, a Hirshfeld surface (HS) analysis was carried out using *Crystal Explorer 17.5* (Spackman *et al.*, 2021 ▸). In the HS plotted over *d*_norm_ (Fig. 3 ▸), the contact distances equal, shorter and longer with respect to the sum of van der Waals radii are shown in white, red and blue, respectively. According to the two-dimensional fingerprint plots, H⋯H, H⋯O/O⋯H, C⋯C and H⋯C/C⋯H contacts make the most important contributions to the HS (Fig. 4 ▸).

## 5. Synthesis and crystallization

Maleic anhydride (61.0 mg, 0.62 mmol) was added to *N*-(3-(thio­phen-3-yl)prop-2-yn­yl)aniline (**1**) (132.3 mg, 0.62 mmol) diluted in PhCH_3_ (3 mL) at a 5 mL round-bottom flask. The resulting mixture was heated under reflux for 5 h, and then cooled to room temperature. The resulting precipitate was filtered, washed with PhMe (3 mL), Et_2_O (2 × 3 mL), and air dried to give acid **2** (20.0 mg, 10%) as a colourless solid (for full characteristics see Shelukho *et al.*, 2025 ▸). After cooling mother liquor at 278 K, the precipitate was filtered, washed with mother liquor (3 mL), Et_2_O (2 × 3 mL), and air dried to give the title compound **3** as yellow plates (yield 49%, 96.4 mg, m.p. > 523 K). IR (KBr), ν (cm^−1^): 1704 (CO_2_), 1591 (N—C=O). ^1^H NMR (700.2 MHz, DMSO-*d*_6_): δ (*J*, Hz) 16.45 (*br.s*., 1H, CO_2_H), 8.45 (*s*, 1H, H-Ar), 8.18 (*d*, *J* = 5.5 Hz, 1H, H-2 Thien), 7.91 (*d*, *J* = 7.6 Hz, 2H, H-Ar), 7.70 (*d*, *J* = 5.5 Hz, 1H, H-Thien), 7.56 (*t*, *J* = 7.6 Hz, 2H, H-Ar), 7.37 (*t*, *J* = 7.6 Hz, 1H, H-Ar), 5.33 (*s*, 2H, NCH_2_) ppm. ^13^C {^1^H} NMR (176.1 MHz, DMSO-*d*_6_): δ 169.1, 165.9, 1447, 142.3, 138.6, 138.1, 136.7, 129.7 (2C), 126.9, 126.8, 123.6, 123.4, 122.6, 122.2 (2C), 52.3 ppm. MS (ESI) *m*/*z*: [*M* + H]^+^ 310. Elemental analysis calculated (%) for C_17_H_11_NO_3_S: C 66.01, H 3.58, N 4.53, S 10.36; found: C 65.84, H 3.49, N 4.69, S 10.17.

## 6. Refinement

Crystal data, data collection and structure refinement details are summarized in Table 2 ▸. The hy­droxy hydrogen atom was located in a difference Fourier map and refined isotropically. The C-bound hydrogen-atom positions were calculated geometrically at distances of 0.95 Å (for aromatic CH) and 0.99 Å (for CH_2_) and refined using a riding model by applying the constraint *U*_iso_(H) = 1.2*U*_eq_(C).

## Supplementary Material

Crystal structure: contains datablock(s) I. DOI: 10.1107/S2056989025008618/ex2095sup1.cif

Structure factors: contains datablock(s) I. DOI: 10.1107/S2056989025008618/ex2095Isup2.hkl

Supporting information file. DOI: 10.1107/S2056989025008618/ex2095Isup3.cml

CCDC reference: 2492514

Additional supporting information:  crystallographic information; 3D view; checkCIF report

## Figures and Tables

**Table 1:** Hydrogen-bond geometry (Å, °)

*D*—H⋯*A*	*D*—H	H⋯*A*	*D*⋯*A*	*D*—H⋯*A*
O3—H3*O*⋯O1	0.74 (8)	1.76 (8)	2.496 (5)	175 (8)
C5—H5*A*⋯O2^i^	0.99	2.37	3.343 (6)	167
C5—H5*B*⋯O2^ii^	0.99	2.37	3.013 (6)	122
C11—H11⋯O1^iii^	0.95	2.50	3.333 (6)	146

Symmetry codes: (i) 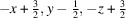; (ii) 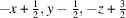; (iii) 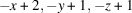.

**Table 2:** Experimental details

Crystal data	
Chemical formula	C_17_H_11_NO_3_S
*M*_r_	309.33
Crystal system, space group	Monoclinic, *P*2_1_/*n*
Temperature (K)	100
*a*, *b*, *c* (Å)	3.89128 (10), 15.8505 (5), 21.6531 (7)
β (°)	93.596 (3)
*V* (Å^3^)	1332.91 (7)
*Z*	4
Radiation type	Cu *K*α
μ (mm^−1^)	2.28
Crystal size (mm)	0.13 × 0.07 × 0.02

Data collection	
Diffractometer	Rigaku XtaLAB Synergy-S, HyPix-6000HE area-detector
Absorption correction	Gaussian (*CrysAlis PRO*; Rigaku OD, 2021 ▸).
*T*_min_, *T*_max_	0.857, 1.000
No. of measured, independent and observed [*I* > 2σ(*I*)] reflections	11945, 2775, 2269
*R*_int_	0.119

Refinement	
*R*[*F*^2^ > 2σ(*F*^2^)], *wR*(*F*^2^), *S*	0.097, 0.238, 1.03
No. of reflections	2775
No. of parameters	203
H-atom treatment	H atoms treated by a mixture of independent and constrained refinement
Δρ_max_, Δρ_min_ (e Å^−3^)	0.94, −0.48

Computer programs: *CrysAlis PRO* (Rigaku OD, 2021 ▸), *SHELXT* (Sheldrick, 2015*a* ▸), *SHELXL* (Sheldrick, 2015*b* ▸) and *SHELXTL* (Sheldrick, 2008 ▸).

## supplementary crystallographic information

### 7-Oxo-6-phenyl-6,7-dihydro-5H-thieno[2,3-f]isoindole-8-carboxylic acid Crystal data

**Table:**

C_17_H_11_NO_3_S	*F*(000) = 640
*M**_r_* = 309.33	*D*_x_ = 1.541 Mg m^−^^3^
Monoclinic, *P*2_1_/*n*	Cu *K*α radiation, λ = 1.54184 Å
*a* = 3.89128 (10) Å	Cell parameters from 3873 reflections
*b* = 15.8505 (5) Å	θ = 3.5–78.8°
*c* = 21.6531 (7) Å	µ = 2.28 mm^−^^1^
β = 93.596 (3)°	*T* = 100 K
*V* = 1332.91 (7) Å^3^	Plate, yellow
*Z* = 4	0.13 × 0.07 × 0.02 mm

### 7-Oxo-6-phenyl-6,7-dihydro-5H-thieno[2,3-f]isoindole-8-carboxylic acid Data collection

**Table:**

Rigaku XtaLAB Synergy-S, HyPix-6000HE area-detector diffractometer	2269 reflections with *I* > 2σ(*I*)
Radiation source: micro-focus sealed X-ray tube	*R*_int_ = 0.119
φ and ω scans	θ_max_ = 79.8°, θ_min_ = 3.5°
Absorption correction: gaussian (CrysAlisPro; Rigaku OD, 2021).	*h* = −3→4
*T*_min_ = 0.857, *T*_max_ = 1.000	*k* = −19→19
11945 measured reflections	*l* = −27→27
2775 independent reflections	

### 7-Oxo-6-phenyl-6,7-dihydro-5H-thieno[2,3-f]isoindole-8-carboxylic acid Refinement

**Table:**

Refinement on *F*^2^	Primary atom site location: difference Fourier map
Least-squares matrix: full	Secondary atom site location: difference Fourier map
*R*[*F*^2^ > 2σ(*F*^2^)] = 0.097	Hydrogen site location: mixed
*wR*(*F*^2^) = 0.238	H atoms treated by a mixture of independent and constrained refinement
*S* = 1.03	*w* = 1/[σ^2^(*F*_o_^2^) + (0.088*P*)^2^ + 6.9*P*] where *P* = (*F*_o_^2^ + 2*F*_c_^2^)/3
2775 reflections	(Δ/σ)_max_ < 0.001
203 parameters	Δρ_max_ = 0.94 e Å^−^^3^
0 restraints	Δρ_min_ = −0.47 e Å^−^^3^

### 7-Oxo-6-phenyl-6,7-dihydro-5H-thieno[2,3-f]isoindole-8-carboxylic acid Special details

**Table:**

Geometry. All esds (except the esd in the dihedral angle between two l.s. planes) are estimated using the full covariance matrix. The cell esds are taken into account individually in the estimation of esds in distances, angles and torsion angles; correlations between esds in cell parameters are only used when they are defined by crystal symmetry. An approximate (isotropic) treatment of cell esds is used for estimating esds involving l.s. planes.

### 7-Oxo-6-phenyl-6,7-dihydro-5H-thieno[2,3-f]isoindole-8-carboxylic acid Fractional atomic coordinates and isotropic or equivalent isotropic displacement parameters (Å^2^)

**Table:**

	*x*	*y*	*z*	*U*_iso_*/*U*_eq_	
S1	0.1382 (3)	0.55415 (8)	0.87334 (5)	0.0250 (3)	
O1	0.7474 (9)	0.5121 (2)	0.63096 (15)	0.0265 (8)	
O2	0.4093 (9)	0.6550 (2)	0.79077 (16)	0.0276 (8)	
O3	0.6243 (11)	0.6318 (2)	0.70111 (19)	0.0332 (9)	
H3O	0.671 (19)	0.596 (5)	0.681 (4)	0.05 (2)*	
C1	0.4827 (12)	0.6057 (3)	0.7506 (2)	0.0239 (10)	
C2	−0.0144 (12)	0.4688 (3)	0.9129 (2)	0.0278 (10)	
H2	−0.1150	0.4741	0.9516	0.033*	
C3	0.0211 (12)	0.3938 (3)	0.8842 (2)	0.0249 (10)	
H3	−0.0494	0.3413	0.9004	0.030*	
C3A	0.1779 (11)	0.4030 (3)	0.8264 (2)	0.0216 (9)	
C4	0.2556 (11)	0.3396 (3)	0.7848 (2)	0.0213 (9)	
H4	0.2094	0.2821	0.7932	0.026*	
C4A	0.4011 (11)	0.3632 (3)	0.7312 (2)	0.0181 (9)	
C5	0.5110 (12)	0.3077 (3)	0.6796 (2)	0.0219 (9)	
H5A	0.6944	0.2680	0.6945	0.026*	
H5B	0.3141	0.2753	0.6607	0.026*	
N6	0.6397 (9)	0.3689 (2)	0.63574 (17)	0.0204 (8)	
C7	0.6339 (12)	0.4491 (3)	0.6577 (2)	0.0218 (9)	
C7A	0.4790 (11)	0.4474 (3)	0.7184 (2)	0.0205 (9)	
C8	0.4125 (11)	0.5128 (3)	0.7595 (2)	0.0209 (9)	
C8A	0.2606 (11)	0.4884 (3)	0.8140 (2)	0.0219 (9)	
C9	0.7463 (11)	0.3427 (3)	0.5766 (2)	0.0213 (9)	
C10	0.7616 (12)	0.3990 (3)	0.5276 (2)	0.0272 (10)	
H10	0.7054	0.4567	0.5330	0.033*	
C11	0.8587 (13)	0.3705 (3)	0.4710 (2)	0.0292 (11)	
H11	0.8698	0.4092	0.4377	0.035*	
C12	0.9398 (12)	0.2871 (4)	0.4619 (2)	0.0297 (11)	
H12	1.0073	0.2684	0.4228	0.036*	
C13	0.9218 (13)	0.2306 (3)	0.5106 (2)	0.0299 (11)	
H13	0.9781	0.1730	0.5046	0.036*	
C14	0.8225 (12)	0.2572 (3)	0.5679 (2)	0.0249 (10)	
H14	0.8065	0.2180	0.6008	0.030*	

### 7-Oxo-6-phenyl-6,7-dihydro-5H-thieno[2,3-f]isoindole-8-carboxylic acid Atomic displacement parameters (Å^2^)

**Table:**

	*U*^11^	*U*^22^	*U*^33^	*U*^12^	*U*^13^	*U*^23^
S1	0.0238 (6)	0.0237 (6)	0.0280 (6)	0.0030 (4)	0.0046 (4)	−0.0064 (4)
O1	0.0360 (19)	0.0144 (16)	0.0301 (17)	−0.0061 (13)	0.0105 (14)	0.0027 (13)
O2	0.0290 (18)	0.0184 (16)	0.0359 (18)	0.0002 (13)	0.0047 (14)	−0.0047 (14)
O3	0.050 (2)	0.0135 (17)	0.038 (2)	−0.0018 (15)	0.0152 (17)	−0.0018 (15)
C1	0.019 (2)	0.019 (2)	0.034 (2)	0.0045 (17)	0.0034 (18)	−0.0033 (18)
C2	0.025 (2)	0.034 (3)	0.025 (2)	0.002 (2)	0.0050 (18)	0.000 (2)
C3	0.021 (2)	0.028 (3)	0.025 (2)	−0.0034 (19)	0.0008 (17)	0.0016 (19)
C3A	0.016 (2)	0.023 (2)	0.026 (2)	0.0028 (17)	0.0013 (16)	−0.0021 (18)
C4	0.024 (2)	0.016 (2)	0.024 (2)	−0.0030 (17)	0.0043 (17)	0.0020 (17)
C4A	0.016 (2)	0.015 (2)	0.024 (2)	−0.0001 (16)	0.0024 (15)	−0.0014 (16)
C5	0.027 (2)	0.015 (2)	0.024 (2)	−0.0025 (17)	0.0071 (17)	0.0010 (17)
N6	0.0211 (19)	0.0150 (18)	0.0255 (19)	−0.0024 (14)	0.0048 (14)	0.0036 (15)
C7	0.023 (2)	0.014 (2)	0.029 (2)	0.0023 (17)	0.0051 (17)	0.0021 (18)
C7A	0.020 (2)	0.017 (2)	0.024 (2)	0.0016 (17)	0.0041 (16)	0.0001 (17)
C8	0.018 (2)	0.020 (2)	0.025 (2)	0.0016 (17)	0.0037 (16)	−0.0037 (17)
C8A	0.014 (2)	0.023 (2)	0.028 (2)	0.0016 (17)	0.0018 (16)	−0.0050 (18)
C9	0.017 (2)	0.023 (2)	0.025 (2)	−0.0015 (17)	0.0050 (16)	−0.0021 (18)
C10	0.028 (3)	0.023 (2)	0.031 (2)	−0.0008 (19)	0.0091 (19)	0.003 (2)
C11	0.023 (2)	0.035 (3)	0.030 (2)	−0.003 (2)	0.0088 (18)	0.002 (2)
C12	0.023 (2)	0.041 (3)	0.026 (2)	−0.005 (2)	0.0077 (18)	−0.003 (2)
C13	0.032 (3)	0.027 (3)	0.031 (3)	0.002 (2)	0.004 (2)	−0.007 (2)
C14	0.023 (2)	0.028 (3)	0.025 (2)	−0.0024 (19)	0.0054 (18)	0.0005 (19)

### 7-Oxo-6-phenyl-6,7-dihydro-5H-thieno[2,3-f]isoindole-8-carboxylic acid Geometric parameters (Å, º)

**Table:**

S1—C2	1.726 (5)	C5—H5A	0.9900
S1—C8A	1.745 (5)	C5—H5B	0.9900
O1—C7	1.249 (6)	N6—C7	1.358 (6)
O2—C1	1.217 (6)	N6—C9	1.431 (6)
O3—C1	1.303 (6)	C7—C7A	1.479 (6)
O3—H3O	0.74 (8)	C7A—C8	1.402 (6)
C1—C8	1.512 (7)	C8—C8A	1.406 (6)
C2—C3	1.353 (7)	C9—C10	1.392 (7)
C2—H2	0.9500	C9—C14	1.402 (7)
C3—C3A	1.434 (6)	C10—C11	1.380 (7)
C3—H3	0.9500	C10—H10	0.9500
C3A—C4	1.396 (6)	C11—C12	1.376 (8)
C3A—C8A	1.420 (7)	C11—H11	0.9500
C4—C4A	1.373 (6)	C12—C13	1.387 (8)
C4—H4	0.9500	C12—H12	0.9500
C4A—C7A	1.400 (6)	C13—C14	1.388 (7)
C4A—C5	1.505 (6)	C13—H13	0.9500
C5—N6	1.467 (6)	C14—H14	0.9500
			
C2—S1—C8A	90.9 (2)	O1—C7—C7A	127.0 (4)
C1—O3—H3O	112 (6)	N6—C7—C7A	108.1 (4)
O2—C1—O3	120.9 (4)	C4A—C7A—C8	121.8 (4)
O2—C1—C8	118.8 (4)	C4A—C7A—C7	107.4 (4)
O3—C1—C8	120.3 (4)	C8—C7A—C7	130.7 (4)
C3—C2—S1	114.4 (4)	C7A—C8—C8A	115.7 (4)
C3—C2—H2	122.8	C7A—C8—C1	126.7 (4)
S1—C2—H2	122.8	C8A—C8—C1	117.7 (4)
C2—C3—C3A	111.9 (4)	C8—C8A—C3A	122.3 (4)
C2—C3—H3	124.1	C8—C8A—S1	127.0 (4)
C3A—C3—H3	124.1	C3A—C8A—S1	110.7 (3)
C4—C3A—C8A	120.1 (4)	C10—C9—C14	119.8 (4)
C4—C3A—C3	127.7 (4)	C10—C9—N6	121.7 (4)
C8A—C3A—C3	112.2 (4)	C14—C9—N6	118.4 (4)
C4A—C4—C3A	117.9 (4)	C11—C10—C9	119.7 (5)
C4A—C4—H4	121.1	C11—C10—H10	120.2
C3A—C4—H4	121.1	C9—C10—H10	120.2
C4—C4A—C7A	122.2 (4)	C12—C11—C10	121.3 (5)
C4—C4A—C5	128.3 (4)	C12—C11—H11	119.4
C7A—C4A—C5	109.5 (4)	C10—C11—H11	119.4
N6—C5—C4A	102.7 (4)	C11—C12—C13	119.2 (5)
N6—C5—H5A	111.2	C11—C12—H12	120.4
C4A—C5—H5A	111.2	C13—C12—H12	120.4
N6—C5—H5B	111.2	C12—C13—C14	120.9 (5)
C4A—C5—H5B	111.2	C12—C13—H13	119.5
H5A—C5—H5B	109.1	C14—C13—H13	119.5
C7—N6—C9	126.7 (4)	C13—C14—C9	119.1 (5)
C7—N6—C5	112.2 (4)	C13—C14—H14	120.4
C9—N6—C5	121.1 (4)	C9—C14—H14	120.4
O1—C7—N6	124.9 (4)		
			
C8A—S1—C2—C3	0.0 (4)	C7—C7A—C8—C1	1.0 (8)
S1—C2—C3—C3A	−0.4 (5)	O2—C1—C8—C7A	179.9 (4)
C2—C3—C3A—C4	179.7 (5)	O3—C1—C8—C7A	−1.2 (7)
C2—C3—C3A—C8A	0.7 (6)	O2—C1—C8—C8A	0.8 (6)
C8A—C3A—C4—C4A	−2.3 (6)	O3—C1—C8—C8A	179.7 (4)
C3—C3A—C4—C4A	178.8 (4)	C7A—C8—C8A—C3A	−0.3 (6)
C3A—C4—C4A—C7A	1.5 (7)	C1—C8—C8A—C3A	178.8 (4)
C3A—C4—C4A—C5	179.4 (4)	C7A—C8—C8A—S1	−178.6 (3)
C4—C4A—C5—N6	179.1 (4)	C1—C8—C8A—S1	0.5 (6)
C7A—C4A—C5—N6	−2.8 (5)	C4—C3A—C8A—C8	1.8 (7)
C4A—C5—N6—C7	3.5 (5)	C3—C3A—C8A—C8	−179.2 (4)
C4A—C5—N6—C9	−174.9 (4)	C4—C3A—C8A—S1	−179.7 (3)
C9—N6—C7—O1	−5.8 (8)	C3—C3A—C8A—S1	−0.6 (5)
C5—N6—C7—O1	175.8 (4)	C2—S1—C8A—C8	178.8 (4)
C9—N6—C7—C7A	175.4 (4)	C2—S1—C8A—C3A	0.3 (3)
C5—N6—C7—C7A	−3.0 (5)	C7—N6—C9—C10	−19.4 (7)
C4—C4A—C7A—C8	−0.1 (7)	C5—N6—C9—C10	158.8 (4)
C5—C4A—C7A—C8	−178.4 (4)	C7—N6—C9—C14	163.3 (4)
C4—C4A—C7A—C7	179.5 (4)	C5—N6—C9—C14	−18.5 (6)
C5—C4A—C7A—C7	1.2 (5)	C14—C9—C10—C11	−1.3 (7)
O1—C7—C7A—C4A	−177.7 (5)	N6—C9—C10—C11	−178.6 (4)
N6—C7—C7A—C4A	1.0 (5)	C9—C10—C11—C12	0.3 (8)
O1—C7—C7A—C8	1.8 (8)	C10—C11—C12—C13	0.3 (8)
N6—C7—C7A—C8	−179.5 (5)	C11—C12—C13—C14	0.2 (8)
C4A—C7A—C8—C8A	−0.5 (6)	C12—C13—C14—C9	−1.2 (7)
C7—C7A—C8—C8A	−179.9 (4)	C10—C9—C14—C13	1.8 (7)
C4A—C7A—C8—C1	−179.6 (4)	N6—C9—C14—C13	179.1 (4)

### 7-Oxo-6-phenyl-6,7-dihydro-5H-thieno[2,3-f]isoindole-8-carboxylic acid Hydrogen-bond geometry (Å, º)

**Table:**

*D*—H···*A*	*D*—H	H···*A*	*D*···*A*	*D*—H···*A*
O3—H3*O*···O1	0.74 (8)	1.76 (8)	2.496 (5)	175 (8)
C5—H5*A*···O2^i^	0.99	2.37	3.343 (6)	167
C5—H5*B*···O2^ii^	0.99	2.37	3.013 (6)	122
C11—H11···O1^iii^	0.95	2.50	3.333 (6)	146

Symmetry codes: (i) −*x*+3/2, *y*−1/2, −*z*+3/2; (ii) −*x*+1/2, *y*−1/2, −*z*+3/2; (iii) −*x*+2, −*y*+1, −*z*+1.
